# Salicylaldehyde Benzoylhydrazones with Anticancer Activity and Selectivity: Design, Synthesis, and In Vitro Evaluation

**DOI:** 10.3390/molecules30051015

**Published:** 2025-02-22

**Authors:** Boryana Nikolova-Mladenova, Rositsa Mihaylova, Mariyana Atanasova, Zvetanka Zhivkova, Irini Doytchinova

**Affiliations:** 1 Department of Chemistry, Faculty of Pharmacy, Medical University of Sofia, 2 Dunav Str., 1000 Sofia, Bulgaria; matanasova@pharmfac.mu-sofia.bg (M.A.); zzhivkova@pharmfac.mu-sofia.bg (Z.Z.); idoytchinova@pharmfac.mu-sofia.bg (I.D.); 2 Department of Pharmacology, Pharmacotherapy and Toxicology, Faculty of Pharmacy, Medical University of Sofia, 2 Dunav Str., 1000 Sofia, Bulgaria; rmihaylova@pharmfac.mu-sofia.bg

**Keywords:** salicylaldehyde, hydrazones, methoxy group, antileukemic activity, selectivity

## Abstract

Dimethoxy derivatives of salicylaldehyde benzoylhydrazone containing a methoxy group on both aromatic rings were designed and synthesized. The compounds were obtained in high yields, and their structures were confirmed by elemental analysis and various spectral techniques. In vitro evaluation of dimethoxy hydrazones demonstrated potent activity against the leukemic cell lines at low micro- and nanomolar concentrations. Remarkably, two dimethoxy analogs showed exceptional antileukemic selectivity, with no toxicity observed in normal human embryonic kidney HEK-293 cells. In silico modeling identified likely interactions with the target, human cAbl kinase, and suggested a possible mechanism for their antileukemic activity.

## 1. Introduction

Cancer remains a formidable challenge in modern medicine, characterized by uncontrolled cell growth, invasion into surrounding tissues, and potential metastasis to distant organs [1,2]. This multifaceted disease encompasses numerous variations, each with unique biological and genetic features, making it a complex health concern worldwide [2]. Advances in cancer research have revolutionized our understanding of the disease’s underlying mechanisms and paved the way for innovative diagnostic and therapeutic strategies [3]. The molecular pathways driving cancer initiation, progression, and resistance to treatments have been elucidated. Efforts in genomics, proteomics, and immunology have been instrumental in identifying specific genetic alterations, biomarkers, and immune-related targets that hold promise for precision medicine and personalized therapies [4]. Immunotherapies, such as immune checkpoint inhibitors [5,6] and CAR-T cell therapies [7], have emerged as groundbreaking approaches, harnessing the body’s immune system to target and eliminate cancer cells selectively. Moreover, the advent of targeted therapies directed against specific molecular alterations in cancer cells has showcased remarkable efficacy in subsets of patients, leading to improved outcomes [8]. In spite of these advancements, tumor heterogeneity and treatment resistance need more precise diagnostic and therapeutic tools [6,9].

Despite the invasion of immunotherapies, small molecules remain pivotal in cancer treatment [10]. Targeted therapies like tyrosine kinase inhibitors (TKIs) disrupt specific signaling pathways crucial for tumor growth [11]. Anti-angiogenic agents inhibit blood vessel formation [12], while hormone therapy drugs target receptors on cancer cells [13]. These molecules impede cancer progression by selectively disrupting key proteins or pathways [14,15]. Combination therapies leveraging small molecules alongside other treatments show promise in combating resistance [16].

Over recent years, our research has centered on investigating the anticancer properties of hydrazones resulting from the fusion of salicylaldehyde derivatives and acyl hydrazides [17,18,19,20,21]. Introducing various substitutions in salicylaldehyde and hydrazide components has slightly modified the original compound while significantly enhancing its pharmacological characteristics. Particularly, the introduction of a methoxy group in the salicylaldehyde structure has led to derivatives exhibiting potent antiproliferative effects. For instance, 3-methoxysalicylaldehyde-derived hydrazones demonstrated strong cytotoxicity against acute myeloid leukemia (AML) HL-60 cells [17]. Notably, incorporating a methoxy group at the fifth position in salicylaldehyde hydrazones significantly heightened activity against the solid breast cancer cell line MCF-7 [18]. These compounds showcased selectivity between malignant and non-tumor cell lines. Further investigations aimed to replace the methoxy group with alternative functional groups to explore their significance. For instance, 5-bromosalicylaldehyde-derived hydrazones displayed heightened activity against T-cell leukemic cell lines SKW-3 and myeloid HL-60 cells [19]. Similarly, 5-nitrosalicylaldehyde benzoylhydrazones exhibited notable cytotoxic activity against leukemic cell lines HL-60 and BV-173 in micromolar concentrations [20]. Recently, we found that the presence of 4-methoxysalicylic moiety, phenyl, and pyridinyl rings significantly increases the antileukemic activity and selectivity [21]. Furthermore, methoxysalicylaldehyde hydrazones can act as excellent ligands [22]. The presence of the methoxy group in hydrazide rings enhances their chelating capability. Dimethoxy hydrazones were synthesized [23,24,25,26] and used as multidentate ligands in oxovanadium(V), oxomolybdenum(VI), copper(II) and ruthenium(II) complexes [27,28,29,30,31,32,33,34]. Their antiglycation activity [25], antimalarial activity [26], and insulin-like activity of the oxovanadium(V) complex [27] were also studied.

Here, we continue to explore the appropriate substituents within the salicylaldehyde benzoylhydrazone scaffold, incorporating methoxy groups in both aromatic rings. In order to evaluate the anticancer potential of dimethoxy hydrazones and the effect of the position of the methoxy group, a series of five dimethoxy derivatives with different positions of the methoxy groups was designed. The compounds were in vitro evaluated against a panel of malignant human cell lines of different tissue origin. Among the five dimethoxy derivatives tested, two exhibited remarkable antileukemic selectivity, demonstrating no toxicity on the normal human embryonic kidney HEK-293 cell line. In silico modeling elucidated the interaction with the probable target human cAbl kinase, shedding light on the possible mechanism of antileukemic activity of the compounds.

## 2. Results and Discussion

### 2.1. Design of Methoxysalicylaldehyde Methoxybenzoylhydrazones and In Silico Drug Likeness Evaluation

In our previous study, it was observed that the average ligand efficiency (LE) of methoxysalicylaldehyde benzoylhydrazones on leukemic cell lines (HL-60, K-562, and BV-173) decreases in the order of 4-methoxy > 3-methoxy > 5-methoxy [21]. Given these observations, we opted to further investigate 4-methoxy and 3-methoxy derivatives, incorporating an additional methoxy group into the phenyl ring of the hydrazide moiety (Table 1).

Prior to synthesis, the designed compounds were evaluated in silico for drug likeness. The screening involved assessing essential physicochemical properties, ADME (absorption, distribution, metabolism, and excretion) characteristics and pharmacokinetic (PK) parameters predicted by previously derived quantitative structure–pharmacokinetics relationship (QSPkR) models [35,36,37]. The calculated values are summarized in Table 2.

The compounds share a molecular weight (*Mw*) of 300.31 g/mol, rendering them more lead-like than drug-like [38]. This lower molecular weight provides an opportunity for additional substitution and structural optimization to enhance anticancer activity and selectivity. The compounds are weak acids with pK_a_ values in the range 8.00–8.50. The pK_a_ values exhibit position dependence, notably with 4-methoxysalicylaldehydes showing higher acidity. At pH 7.4, the fraction of ionized molecules f_A_ reaches approximately 10% for 3-methoxysalicylaldehydes and 20% for 4-methoxysalicylaldehydes. With logP values between 3.31 and 3.86, the compounds again are positioned more as lead-like than drug-like. An 80 Å polar surface area (PSA) aligns with both lead- and drug-likeness criteria. Despite having six free rotatable bonds, these molecules exhibit rigidity due to p–π conjugation between aromatic rings and the hydrazone moiety. They possess two hydrogen bond donors and six acceptors and fully comply with Lipinski’s rule.

Concerning ADME properties, dimethoxy hydrazones demonstrate moderate water solubility, high gastrointestinal (GI) absorbability, with a 55% probability of exceeding 10% bioavailability in rats (BA score) attributed to the low fraction of Csp3 atoms, and they exhibit no blood–brain barrier (BBB) permeability. Compounds **3** and **4** likely act as inhibitors of CYP1A2, with compound **4** additionally inhibiting CYP2C9. Neither compound functions as a substrate of P-gp. Indeed, all compounds exhibit characteristics of both lead- and drug-likeness. Their synthetic accessibility, ranging between 2.62 and 2.72, indicates a relatively straightforward synthesis.

Regarding the predicted PK parameters [35,36,37], all compounds exhibit low steady-state volumes of distribution (VD_ss_) (0.20–0.21 L/kg), aligning closely with the volume of extravascular body water. The notably low fraction of the unbound to plasma protein molecule *f_u_* suggests extensive binding to plasma proteins of almost 99%. Acidic drugs typically bind strongly to human serum albumin [39]. The total clearance (CL) is between 0.39 and 0.45 L/h/kg and classifies these compounds as low- to moderate-CL compounds. An analysis of 754 drugs in various ionization states revealed that 78% of anionic drugs exhibit low CL (<0.24 L/h/kg), with only 1–2% exhibiting high CL (>0.96 L/h/kg) [40]. The short half-life of 0.32–0.37 h stems from the low VD_ss_ and CL values.

The in silico screening for drug likeness of the designed methoxysalicylaldehyde methoxybenzoylhydrazones revealed their promising potential, encouraging us to proceed with their synthesis.

### 2.2. Synthesis of Dimethoxy Hydrazones

**Scheme 1:**
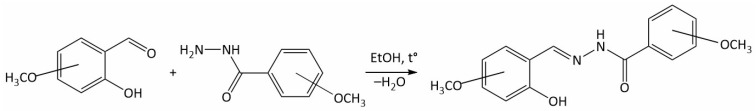
Synthesis of dimethoxy hydrazones.

The series of dimethoxy hydrazones were synthesized in one step by Schiff base condensation in ethanol between 3-methoxy- or 4-methoxysalicylaldehyde and appropriate hydrazides—2-methoxy-, 3-methoxy- or 4-methoxybenzhydrazide—according to a scheme (Scheme 1) previously described [17,18,19,20,21].

The dimethoxy hydrazones were obtained in high yields, and their molecular formulas were confirmed by elemental analysis. IR, ^1^H NMR, and ^13^C NMR spectroscopy determined the structures of the compounds. The characteristic absorption band around 1600–1609 cm^−1^ in the IR spectra of the hydrazones, assigned to C=N imino stretching vibrations, confirmed the condensation between the aldehyde group of methoxysalicylaldehydes and the amino group of methoxyhydrazides and formation of the Schiff bases. The medium intensity peaks around 3324–3450 cm^−1^ and 3192–3295 cm^−1^ corresponded to the phenolic OH and NH groups, respectively. The intensive sharp band at 1739–1747 cm^−1^ was due to the frequency vibration of the carbonyl group C=O and indicated the keto form of the dimethoxy hydrazones in the solid state. The hydrazones were further studied by their ^1^H NMR and ^13^C NMR spectra in deuterated dimethyl sulfoxide (DMSO-d_6_). Signals for the protons characteristic of the hydrazone azomethine group HC=N- were observed at 8.53–8.63 ppm in the ^1^H NMR spectra. The broad singlets around 11.66–12.04 ppm were assigned to the protons of the phenolic OH group from the aldehyde ring and broad singlets at 10.96–11.71 ppm were due to the protons of the NH group. The protons of the two methoxy groups appeared as singlets at 3.78–3.89 ppm. ^13^C NMR spectra provided additional information about the carbon atoms in the molecules of dimethoxy hydrazones. The signals corresponding to the carbon atoms of the azomethine group appeared at 147.93–148.88 ppm. The peaks at 162.11–162.57 ppm were due to the C=O group. The signals for carbon atoms of the methoxy groups were located at 55.32–55.85 ppm.

### 2.3. In Vitro Anticancer Activity and Selectivity

The anticancer activity of the dimethoxy hydrazone derivatives was tested against a panel of malignant human cell lines of different tissue origin and molecular characteristics, including several in vitro leukemia models (acute promyelocytic leukemia HL-60; bcr-abl+ chronic myeloid leukemia cell lines BV-173, K-562, and AR-230; and T-cell leukemia SKW-3), as well as the two major phenotypes of human breast adenocarcinoma (hormone-sensitive MCF-7 cells with high progesterone (PR) and estrogen receptor α (ERα) expression and triple-negative breast adenocarcinoma (TNBC) MDA-MB-231 lacking ERα, PR, and human epidermal growth factor receptor 2 (HER2)). The existence and degree of cancer selectivity of the tested compounds were evaluated against normal human embryonic kidney HEK-293 cells. The calculated IC_50_ values are summarized in Table 3.

As evident from the cytotoxicity data presented, all compounds in the series exerted markedly stronger growth-inhibitory effects against the suspension cell cultures of leukemic origin (HL-60, BV-173, K-562, AR-230, and SKW-3) at low micro- and nanomolar concentrations, with the only exception being compound **1** (3-methoxysalicylaldehyde-2-methoxybenzoylhydrazone). The overall antileukemic activity of the latter appeared to be 5 to more than 10 folds lower compared to the other derivatives; however, its efficacy against the epithelial breast carcinoma models was consistent with the entire series. Accordingly, the estimated IC_50_ values for both the MCF-7 and MDA-MB-231 cell lines were at least one order of magnitude higher than the half-inhibitory concentrations against the leukemic models for all five hydrazone derivatives. Furthermore, all compounds exhibited similar in vitro effects on both types of breast cancer, ruling out any relevance of their phenotypic characteristics to their chemosensitivity profile and any involvement of the hormone (ER, PR) and HER2 receptors in the molecular mechanisms of cytotoxicity. The variations in activity according to cell type (leukemic versus epithelial and suspension versus adherent) are also seen in the cytotoxicity profiles of the compounds against the other epithelial culture HEK-293, which resembled those of the carcinoma MCF-7 and MDA-MB-231 cell lines. Moreover, compounds **1** and **3** showed negligible antiproliferative activity towards the healthy HEK-293 cells used as reference (IC_50_ >100 µM), accounting for their remarkable tumor selectivity (Table 3). The calculated selectivity ratios for the structural isomers **2**, **4**, and **5** were not as favorable; however, they still indicate a strong preferential inhibition of the malignant leukemic cultures over the normal HEK-293 cells. The highest chemosensitivity to the tested compounds was observed in the SKW-3 T-cell leukemia model, where the most potent analogs, analogs **4** and **5**, had IC_50_ values below 1 µM, and the least active representative **1** aberrantly produced antileukemic activity (IC_50_ 4.3 µM) similar to that of the other members of the series.

The ligand efficiencies (LEs), obtained by the ratio between pIC_50_ and the number of heavy atoms in the molecule, along with the selectivity indices (SIs), calculated by comparing IC_50_ of normal cells to IC_50_ of cancer cells, are presented in Table 4.

The average LEs of compounds on leukemic cells range between 0.216 and 0.266, surpassing the LEs observed of compounds on breast cancer cells. The LEs of compounds on leukemic cells decrease in the order **5** > **4** > **3** > **2** > **1**. Surprisingly, compound **3** is not active on the AR-230 cell line. Compounds **1** and **3** exhibit no toxicity on normal HEK-293 cells. Compound **3** demonstrates an SI > 42 on HL-60, >37 on SKW-3, >36 on K-562, >19 on BV-173, and the highest average SI (>27). The highest SIs are shown in the SKW-3 cell line (between 11 and >37).

The anticancer activities and selectivity observed in the present study reveal compound **3** (3-methoxysalicylaldehyde-4-methoxybenzoylhydrazone) as the most promising analog of the series.

### 2.4. In Silico Modeling of the Interactions Between the Methoxy Salicylaldehyde Methoxybenzoylhydrazones and Human cAbl Tyrosine Kinase

Compounds **2**–**5** showed high activity on BV-173 and K-562 cells. These cells are known to express the BCR-ABL1 fusion protein which is a hallmark of chronic myeloid leukemia (CML) and is associated with abnormal proliferation and survival of myeloid cells [41,42,43]. It results from a translocation between chromosomes 9 and 22, known as the Philadelphia chromosome (t(9;22)(q34;q11)), which fuses the BCR (breakpoint cluster region) gene on chromosome 22 with the ABL1 (Abelson murine leukemia viral oncogene homolog 1) gene on chromosome 9 [41,42,43]. This fusion gene produces a chimeric protein with constitutive tyrosine kinase activity, leading to dysregulated signaling pathways and leukemic transformation.

We hypothesized that BCR-ABL1 fusion protein could be a probable target of the methoxysalicylaldehyde methoxybenzoylhydrazones. To explore this hypothesis, we modeled in silico the interactions between the compounds and human cAbl tyrosine kinase (cAbl TK) by molecular docking and molecular dynamics (MD) simulations.

**Figure 1:**
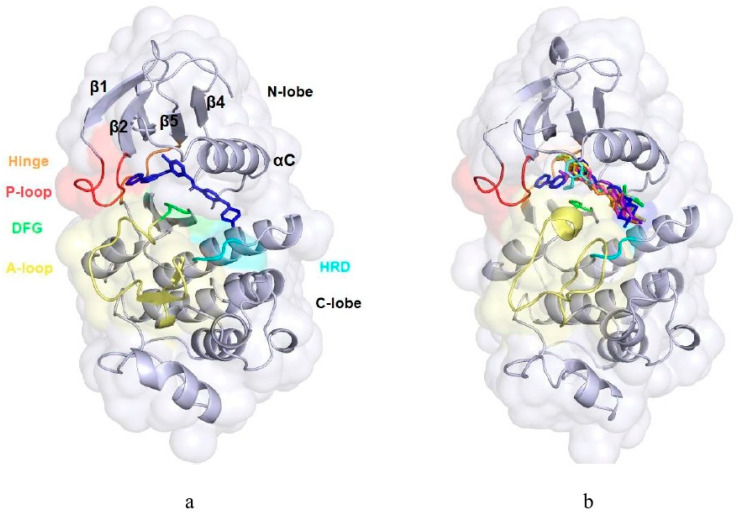
(**a**) The ATP binding site of cAbl tyrosine kinase in complex with imatinib (PDB ID: 2HYY) [46]. The main segments are depicted as follows: the P-loop in red, the hinge region in orange, the HRD motif in cyan, and the DFG motif in green, which is located at the beginning of the A-loop, represented in yellow. The molecule of imatinib is presented in dark blue. (**b**) The most populated poses of imatinib (in dark blue; at 749.4 ns), compound 1 (in pale blue; at 642.9 ns), compound 2 (in magenta; at 556.2 ns), compound 3 (in green; at 752.1 ns), compound 4 (in purple; at 218.4 ns), and compound 5 (in yellow; at 801.3 ns) within the ATP binding site of Abl TK are superimposed over the Abl TK conformation of the complex with imatinib.

The ATP BS of human cAbl TK is a deep hydrophobic pocket located in the cleft between the N-lobe and C-lobe (Figure 1). The smaller N-lobe consists of five β-strands (β1–β5) and one αC-helix (αC), while the larger C-lobe is mostly α-helical. These lobes are flexibly connected via the hinge region, a linker responsible for the opening and closing of the kinase domain [44]. The ATP binding site is enclosed by several key segments, including the P-loop, αC-helix, hinge region, HRD motif, and DFG motif (Figure 1). The P-loop, or phosphate-binding loop, is positioned between the β1- and β2-strands. The αC-helix serves as an allosteric structural component, essential for maintaining the active conformation of the kinase. It is part of the regulatory R-spine core, a stacked configuration of four hydrophobic residues (RS1–RS4), with Met290 acting as RS3. In the active state of Abl TK, Glu286 from the αC-helix forms a salt bridge with Lys271 from β3. The HRD motif (His361-Arg362-Asp363), located in the catalytic loop, stabilizes the active site (activation loop, A-loop) and is involved in ATP binding. The DFG motif (Asp381-Phe382-Gly383) is at the N-terminus of the A-loop and plays a key role in orienting the A-loop, acting as a pivot. Additionally, the aspartic acid in the DFG motif coordinates a magnesium ion, critical for substrate binding and phosphate transfer [44,45]. In the complex with imatinib, depicted in Figure 1, the DFG motif adopts a flipped position, known as the DFG-out conformation, while the αC-helix remains in the active position (C-in). This is supported by the occupation of the RS3 pocket by Met290 (RS3) and the intact salt bridge between Glu286 and Lys271.

The dimethoxy hydrazones were docked into the crystallographic structure of human cAbl TK (PDB ID: 2HYY) [46]. The scoring function ChemPLP scores are presented in Table 5. The tested compounds showed lower docking scores compared to imatinib, primarily due to their significantly lower molecular weight (300.31 g/mol vs. 493.60 g/mol for imatinib). However, when recalculated per unit weight, the scores of the dimethoxy hydrazones became comparable to those of imatinib. Low-molecular-weight starting points, along with maintaining low molecular weight during optimization, are critical aspects of medicinal chemistry in lead-like drug discovery [47,48].

**Figure 2:**
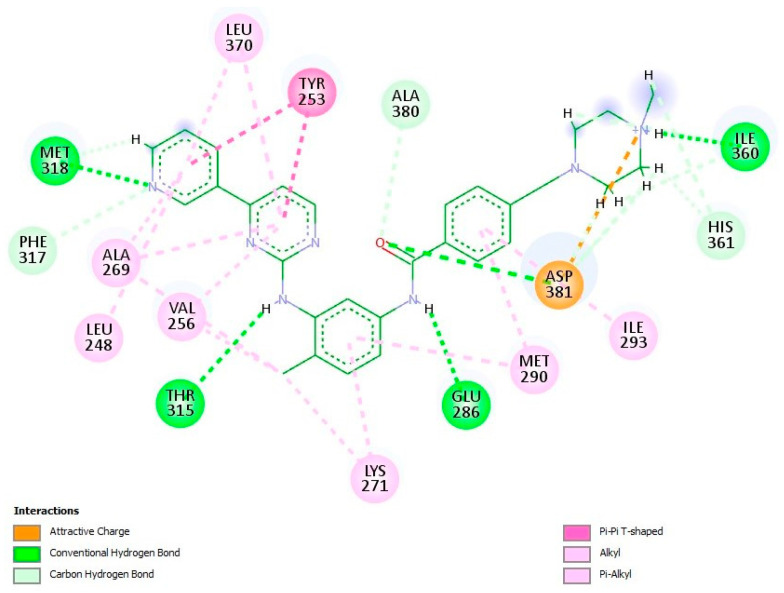
Intermolecular interactions of the representative frames of the most populated clusters resulting from MD simulations of imatinib (in 749.4 ns) within the ATP BS of Abl TK. This 2D diagram was generated utilizing Biovia Discovery Studio Visualizer v21.1.0.20298 [49].

**Figure 3:**
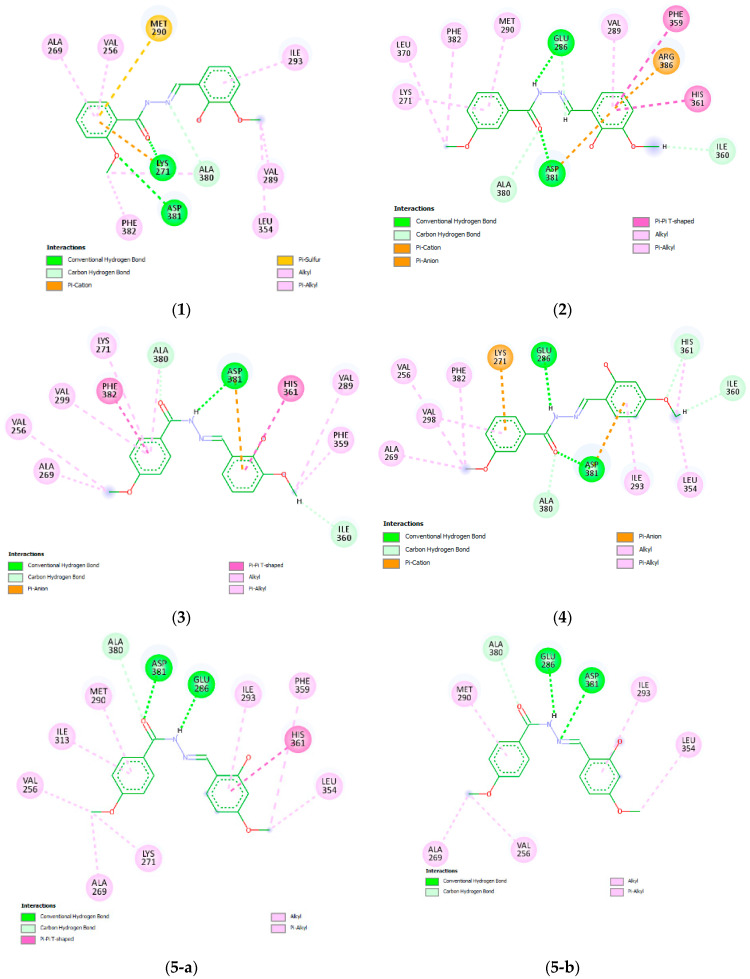
Intermolecular interactions of the representative frames of the most populated clusters resulting from MD simulations of compound **1** (in 642.9 ns), compound **2** (in 556.2 ns), compound **3** (in 752.1 ns), compound **4** (in 218.4 ns), compound **5-a** (in 801.3 ns), and compound **5-b** (in 358.5 ns) within the ATP BS of Abl TK. These 2D diagrams were generated utilizing Biovia Discovery Studio Visualizer v21.1.0.20298 [49].

The docked complexes with the highest scores (Figure S1, Supplementary Materials) were further assessed for stability using molecular dynamics (MD) simulations. The root-mean-square deviation (RMSD) and root-mean-square fluctuation (RMSF) values for the αC-helix protein atoms and the ligands’ heavy atoms are shown in Figure S2. The RMSD values for the αC-helix protein atoms in the systems fluctuated between 1.5 and 2.5 Å. However, in the case of compound **3**, during the final 300 ns of the simulation, the values increased slightly to 3.5–4 Å. Detailed analyses of the αC-helix protein atom fluctuations (RMSFs) are also presented in Figure S2. The RMSD values for the ligands’ heavy atoms (Figure S2, bottom) indicate that all ligands remained stable within the ATP BS throughout the 1 µs MD simulation. Additionally, hierarchical agglomerative clustering of the 1 µs production dynamics trajectories was performed, revealing the most populated clusters for the studied compounds. Representative frames of the most populated clusters for the Abl TK complexes with compounds **1**–**5**, along with the reference compound imatinib, are depicted in Figure 2 and Figure 3.

The most populated cluster in the complex with imatinib consists of 3180 frames out of a total of 3333, accounting for 95.5%. The intermolecular interactions within this cluster are given in Figure 2. Imatinib forms five hydrogen bonds within the ATP BS of the Abl TK: between the pyridine nitrogen atom and the backbone NH group of Met318 in the hinge region; between the anilino NH donor group and the oxygen atom of the side chain OH group of the gatekeeper residue Thr315 at β5; between the amide NH donor group and the carboxyl group of Glu286 from the αC-helix; between the amide CO acceptor group and the backbone NH group of Asp381 from the DFG motif; and between the protonated N-methylpiperazine group and the backbone carbonyl group of Ile260, which precedes the HRD motif in the catalytic loop. Additionally, the negatively charged side chain of Asp381 from the DFG motif is attracted to the positively charged N-methylpiperazine group. The orientation of Tyr253 in the P-loop is critical for enabling π–π stacking interactions with the pyridine and pyrimidine rings of the inhibitor.

The most populated cluster of compound **1** in complex with Abl TK consists of 1646 frames out of a total of 3333, accounting for 49% of the simulation. This indicates that for nearly half of the simulation time (~500 ns), the ligand and residues within a 5 Å radius of the binding site (BS) (as described in Section 3.5 of Materials and Methods) maintained this conformation. The intermolecular interactions in the representative frame from this cluster, at 642.9 ns, are shown in Figure 3. Compound **1** forms three hydrogen bonds: two between the hydrogen atoms of the terminal amino group of Lys271 and the oxygen atom of the carbonyl group in the linker and a third between the backbone NH group of Asp381 from the DFG motif and the oxygen atom of the methoxy substituent in the benzoyl moiety of compound **1**. The position of the positively charged terminal amino group of Lys271 facilitates a cation–π interaction with the benzoyl ring. Additionally, the orientation of the sulfur atom of Met290 in the αC-helix of the N-lobe supports a sulfur–π interaction with the π-conjugated system of the benzoyl group. Numerous van der Waals interactions are also formed within the BS.

The most populated cluster of the complex with compound **2** comprises 2302 frames out of 3333, corresponding to 69% of the total simulation time. The intermolecular interactions are illustrated in the representative frame at 556.2 ns (Figure 3). Two hydrogen bonds are formed: one between the NH donor group of the spacer and the carboxyl group of Glu286 from the αC-helix in the N-lobe and the other between the carbonyl oxygen atom of the linker and the hydrogen atom of the backbone NH group of Asp381 from the DFG motif in the A-loop. The positions of the terminal charged groups of Asp381 and Arg386 in the A-loop facilitate the formation of anion–π and cation–π interactions, respectively, with the salicylaldehyde moiety. Additionally, the conformations of Phe359 and His361 in the HRD motif at the catalytic loop are conducive to T-shaped π–π stacking with the salicylaldehyde moiety.

In the complex with compound **3**, the most populated cluster consists of 2600 frames, representing 78% of the total MD frames. The intermolecular interactions at 752.1 ns are shown in Figure 3. A hydrogen bond is formed between the NH donor group of the linker and the backbone carbonyl oxygen atom of Asp381 from the DFG motif. The negatively charged side chain of Asp381 is positioned for an anion–π interaction with the salicylaldehyde moiety. His361 from the HRD motif in the catalytic loop is suitably oriented for π–π stacking with the same aromatic group. Additionally, the position of Phe382 (RS2) from the DFG motif in the A-loop facilitates π–π stacking with the benzoyl moiety of compound **3**.

The intermolecular interactions of compound **4** at 218.4 ns, the representative frame of the most populated cluster consisting of 3046 frames (91.4%), are shown in Figure 3. This compound is one of the most active against the K-652 cell line (IC_50_ = 1.6 µM, Table 3). Notably, two intramolecular hydrogen bonds stabilize the conformation of the most populated pose, reducing the molecule’s flexibility. These hydrogen bonds occur between the unsaturated nitrogen atom of the hydrazone moiety, the carbonyl oxygen atom from the linker, and the hydrogen atom of the hydroxyl group on the salicylaldehyde ring (Figure S3). This observation supports the idea that a more rigid active analog exhibits higher activity, as it undergoes less conformational entropy change during interaction with the protein. Two intermolecular hydrogen bonds are also formed: one between the carbonyl oxygen atom and the backbone nitrogen atom of Asp381 from the DFG motif and another between the hydrogen atom of the hydrazone group and the carbonyl side chain of Glu286 from the αC-helix. The orientations of both π-aromatic systems in compound **4**, and the benzoyl and salicylaldehyde rings, facilitate the formation of cation–π and anion–π interactions with Lys271 in β3 and Asp381, respectively.

The complex of Abl TK with compound **5** exhibited the most potent activity against the K-562 cell line, and the system displayed two nearly equally populated poses. The first cluster encompassed 1459 frames (43.8%), while the second cluster comprised 1401 frames (42%). The representative frames at 801.3 ns for the first cluster and at 358.5 ns for the second cluster are shown in Figure 3. In the first pose, compound **5** is stabilized similarly to compound **4** through intramolecular hydrogen bonds (Figure S3). Both analogs form the same intermolecular hydrogen bonds, as they differ only in the position of the R2 methoxy substituent on the phenyl ring. However, this slight difference likely shifts the salicylaldehyde ring toward His361 in the HRD motif at the catalytic spine, leading to π–π T-shaped stacking and the loss of both cation–π and anion–π interactions. In the second pose at 358.5 ns, two intermolecular hydrogen bonds stabilize the complex (Figure 3). These are between both hydrazone nitrogen atoms of the linker and Glu286 at the αC-helix, and Asp381 from the DFG motif in the A-loop. Numerous hydrophobic interactions further enhance the stability of the complexes.

It is noteworthy that compounds with similar activity levels against the K-562 cell line, such as compounds **2**, **3**, **4**, and **5** (Table 3), all form hydrogen bonds between the carbonyl (CO) and amino (NH) groups of the linker and Asp381 from the DFG motif in the A-loop, as well as Glu286 from the αC-helix. However, the most selective compound **3** (Table 4), is engaged in two π–π stacking interactions between its aromatic rings (phenyl and salicylaldehyde) and Phe382, the RS2 residue from the DFG motif in the A-loop, and His361 from the HRD motif in the catalytic loop.

These findings will be further explored through in vitro assays with Abl TK, and additional structure optimization will be conducted to discover more potent and selective Abl TK inhibitors.

## 3. Materials and Methods

### 3.1. Materials

All chemicals utilized were of analytical reagent grade. 3-methoxy-, 4-methoxysalicylaldehyde, 2-methoxy-, 3-methoxy-, and 4-methoxybenzhydrazide and 96% ethanol were provided by Merck (Darmstadt, Germany). The compounds’ carbon, nitrogen, and hydrogen contents were evaluated by elemental analyses on a Euro EA 3000-Single, EuroVector SpA (Milan, Italy). The melting points were determined using a Buchi B-540 apparatus (Flawil, Switzerland). Infrared spectra were recorded on a Nicolet iS10 FT-IR spectrometer with a Smart iTR adapter. The ^1^H and ^13^C NMR spectra were acquired on a Bruker Avance 400 NMR spectrometer (Rheinstetten, Germany) in DMSO-d_6_ as a solvent and tetramethylsilane (TMS) as an internal standard. Chemical shifts (δ) were reported in parts per million (ppm); J values were given in Hz. Splitting patterns were denoted by the following symbols: s (singlet), d (doublet), t (triplet), and m (multiplet).

### 3.2. Characterization of Dimethoxy Hydrazones

Dimethoxy hydrazones were synthesized by the reaction of Schiff base condensation in ethanol, as previously described [17,18,19,20,21]

N′-(2-hydroxy-3-methoxybenzylidene)-2-methoxybenzohydrazide (**1**)

Yield: 82%; m.p.: 99–101 °C; color: pale yellow; IR (ν cm^−1^): 3439 (OH), 3262 (NH), 1747 (C=O), 1605 (C=N), 1574 (C-NH). ^1^H NMR (400 MHz, DMSO-d_6_) δ ppm: 3.82 (s, 3H, OCH_3_), 3.89 (s, 3H, OCH_3_), 6.86 (t, 1H, *J* = 7.9 Hz), 7.07 (m, 3H), 7.18 (d, 1H, *J* = 8.5 Hz), 7.51 (m, 1H), 7.65 (dd, 1H, *J* = 7.6, 1.8 Hz), 8.55 (s, 1H, CH=N), 10.99 (s, 1H, NH), 11.66 (s, 1H, OH). ^13^C NMR (101 MHz, DMSO-d_6_) δ ppm: 56.30 (2OCH_3_), 112.44, 114.29, 119.31, 119.51, 120.80, 121.01, 121.38, 123.33, 130.49, 132.92, 147.63, 148.40, 157.25, 162.57 (C=O). Calculated for C_16_H_16_N_2_O_4_: C, 63.99; H, 5.37; N, 9.33. Found: C, 64.11; H, 5.13; N, 9.46.

N′-(2-hydroxy-3-methoxybenzylidene)-3-methoxybenzohydrazide (**2**)

Yield: 85%; m.p.: 115–117 °C; color: pale yellow; IR (ν cm^−1^): 3367 (OH), 3192 (NH), 1739 (C=O), 1609 (C=N), 1562 (C-NH). ^1^H NMR (400 MHz, DMSO-d_6_) δ ppm: 3.82 (s, 3H, OCH_3_), 3.84 (s, 3H, OCH_3_), 6.87 (t, 1H, *J* = 7.9 Hz), 7.04 (dd, 1H, *J* = 8.1, 1.3 Hz), 7.17 (m, 2H), 7.46 (m, 2H), 7.52 (d, 1H, *J* = 7.7 Hz), 8.66 (s, 1H, CH=N), 10.96 (s, 1H, NH), 12.04 (s, 1H, OH). ^13^C NMR (101 MHz, DMSO-d_6_) δ ppm: 55.84 (OCH_3_), 56.29 (OCH_3_), 113.32, 114.30, 118.20, 119.39, 119.53, 120.30, 121.25, 130.23, 134.69, 147.63, 148.42, 148.70, 159.73, 163.02 (C=O). Calculated for C_16_H_16_N_2_O_4_: C, 63.99; H, 5.37; N, 9.33. Found: C, 64.26; H, 5.21; N, 9.28.

N′-(2-hydroxy-3-methoxybenzylidene)-4-methoxybenzohydrazide (**3**)

Yield: 78%; m.p.: 146–148 °C; color: pale yellow; IR (ν cm^−1^): 3324 (OH), 3209 (NH), 1739 (C=O), 1605 (C=N), 1574 (C-NH). ^1^H NMR (400 MHz, DMSO-d_6_) δ ppm: 3.81 (s, 3H, OCH_3_), 3.84 (s, 3H, OCH_3_), 6.86 (t, 1H, *J* = 7.9 Hz), 7.03 (dd, 1H, *J* = 8.0, 1.01 Hz), 7.07 (m, 2H), 7.12 (dd, 1H, *J* = 7.9, 1.3 Hz), 7.93 (d, 2H, *J* = 8.6 Hz), 8.63 (s, 1H, CH=N), 11.09 (s, 1H, NH), 11.97 (s, 1H, OH). ^13^C NMR (101 MHz, DMSO-d_6_) δ ppm: 55.93 (OCH_3_), 56.28 (OCH_3_), 114.22, 114.30, 119.30, 119.46, 121.40, 125.28, 130.05, 147.63, 148.21, 148.40, 162.67, 162.67 (C=O). Calculated for C_16_H_16_N_2_O_4_: C, 63.99; H, 5.37; N, 9.33. Found: C, 64.28; H, 5.19; N, 9.27.

N′-(2-hydroxy-4-methoxybenzylidene)-3-methoxybenzohydrazide (**4**)

Yield: 84%; m.p.: 106–108 °C; color: pale yellow; IR (ν cm^−1^): 3358 (OH), 3215 (NH), 1742 (C=O), 1600 (C=N), 1574 (C-NH). ^1^H NMR (400 MHz, DMSO-d_6_) δ ppm: 3.78 (s, 3H, OCH_3_), 3.84 (s, 3H, OCH_3_), 6.52 (m, 2H), 7.17 (m, 1H), 7.43 (m, 1H), 7.46 (m, 2H), 7.51 (d, 1H, *J* = 7.6 Hz), 8.55 (s, 1H, CH=N), 11.62 (s, 1H, NH), 11.96 (s, 1H, OH). ^13^C NMR (101 MHz, DMSO-d_6_) δ ppm: 55.80 (2OCH_3_), 101.65, 106.97, 112.23, 113.30, 118.07, 120.23, 130.20, 131.62, 134.76, 149.34, 159.72, 159.91, 162.57, 162.81 (C=O). Calculated for C_16_H_16_N_2_O_4_: C, 63.99; H, 5.37; N, 9.33. Found: C, 64.19; H, 5.30; N, 9.23.

N′-(2-hydroxy-4-methoxybenzylidene)-4-methoxybenzohydrazide (**5**)

Yield: 79%; m.p.: 185–187 °C; color: pale yellow; IR (ν cm^−1^): 3450 (OH), 3295 (NH), 1741 (C=O), 1607 (C=N), 1574 (C-NH). ^1^H NMR (400 MHz, DMSO-d_6_) δ ppm: 3.78 (s, 3H, OCH_3_), 3.84 (s, 3H, OCH_3_), 6.51 (m, 2H), 7.07 (m, 2H), 7.40 (d, 1H, *J* = 8.5 Hz), 7.92 (d, 2H, *J* = 8.7 Hz), 8.53 (s, 1H, CH=N), 11.71 (s, 1H, NH), 11.88 (s, 1H, OH). ^13^C NMR (101 MHz, DMSO-d_6_) δ ppm: 55.78 (OCH_3_), 55.91 (OCH_3_), 101.65, 106.89, 112.28, 114.27, 125.35, 129.96, 131.65, 148.80, 159.88, 162.43, 162.50, 162.59 (C=O). Calculated for C_16_H_16_N_2_O_4_: C, 63.99; H, 5.37; N, 9.33. Found: C, 64.25; H, 5.26; N, 9.44.

### 3.3. Cell Lines and Culture Conditions

The spectrum of anti-tumor activity of the experimental compounds was assessed against human malignant cell lines of leukemic (HL-60, BV-173, K-562, AR-230, and SKW-3) and epithelial (MCF-7 and MDA-MB-231) origin, as well as normal human embryonic kidney cells (HEK-293). All cell lines were purchased from the German Collection of Microorganisms and Cell Cultures (DSMZ GmbH, Braunschweig, Germany), and cell cultures were maintained and grown in the appropriate medium specified by the provider and incubated under standard conditions of 37 °C and 5% humidified CO_2_ atmosphere.

### 3.4. Mosmann’s MTT Test for Assessing Cell Viability

The cytotoxicity assessment of the methoxysalicylaldehyde methoxybenzoylhydrazone series was performed using the Mosmann MTT method, a validated technique for evaluating cell viability [50]. Cells in the exponential growth phase were harvested and seeded (100 µL/well) in 96-well plates at a suitable density (3 × 10^5^ cells/well for suspension leukemic cultures and 1.5 × 10^5^ cells/well for adherent epithelial cells). After a 24 h incubation period, the cells were treated with serial dilutions of the tested compounds within an appropriate concentration range. Following a 72 h exposure, an MTT substrate solution (5 mg/mL in PBS) was added to each well. After an additional 4 h incubation, purple insoluble formazan crystals formed. These crystals were then dissolved in an isopropyl alcohol solution containing 5% formic acid before measuring absorbance at 550 nm using a microplate reader Labexim LMR-1 (Vienna, Austria). The recorded absorbance values were blanked against MTT and isopropanol solution and normalized to the mean value of the untreated control (representing 100% cell viability). The experimental data were analyzed using non-linear regression analysis in GraphPad Prism^®^ software 8.0 (San Diego, CA, USA), from which semi-logarithmic “dose–response” curves were constructed, and the half-inhibitory concentrations (IC_50_) of the tested compounds were calculated.

### 3.5. In Silico Evaluation, Molecular Docking, and Molecular Dynamics

The studied molecules were modeled via Discovery Studio Visualizer v21.1.0.20298 [49] and were subject to molecular docking to the X-ray structure of human Abelson (Abl) tyrosine kinase (PDB ID: 2HYY) [46]. The calculations were performed via GOLD software v.5.2.2 (CCDC Ltd., Cambridge, UK) [51] using the following settings: scoring function: ChemPLP; search efficiency: 100%; GA runs: 10; binding site (BS) was defined in 6 Å radius from the original ligand, imatinib; four crystal water molecules (HOH 601, HOH602, HOH603, and HOH604) were set to toggle and spin; protein residues from the BS were kept rigid, while ligands’ rotatable bonds were flexible. The protocol was validated through the RMSD value of the re-docked original ligand from the crystallographic structure, imatinib, which was 0.4634 Å. The top-scored poses of the compounds within the BS of Abl tyrosine kinase were further simulated via molecular dynamics. Molecular dynamic simulations (MDSs) were performed using Amber 18 package (UCSF, San Francisco, CA, USA) [52] as described previously [53]. Briefly, chain A forms the X-ray structure with PDB ID: 2HYY was used as the missing glutamate residue (E275) and was modeled in with MODELLER 9.25 (UCSF, San Francisco, CA, USA) [54]; N and C termini were capped with ACE and NME residues, respectively; GAFF 2.11 force field [55] and AM1-BCC charges [56] were used to derive ligands’ parameters. The systems were sequentially solvated using the TIP3P water model [57] with saline in a truncated octahedral box with a minimum wall distance of 13.5 Å; minimized in two steps, i.e., initially applying the steepest descent method with restraints on all heavy atoms, followed by conjugate gradient minimization; then heated to 300 K for 1 ns at constant volume with heavy atoms restraints; equilibrated at constant pressure density with restraints; pre-production-dynamics-equilibrated without restraints in the NPT ensemble; and finally simulated for 1000 ns (1 µs) using the ff14SB force field [58] under periodic boundary conditions. The cutoff for van der Waals and electrostatic interactions was 12.0 Å. The long-range electrostatics were calculated via the particle mesh Ewald (PME) scheme [59]. The SHAKE algorithm [60] was applied to the covalent bonds to hydrogen atoms. The time step was 2 fs, and frames were saved every 0.3 ns; there were 3333 in total per trajectory. The post-processing analyses, including RMSDs, RMSFs, and clustering, were performed using cpptraj v4.14.0 [61]. We performed hierarchical agglomerative clustering on the production run frames using the ligand and all protein residues within 5 Å of the ligand in our starting structure for the clustering. It was shown that the monoprotonated form of imatinib is bound to the ATP BS [62,63,64]. Therefore, the protonated N-methylpiperazine group was used in the simulations.

## 4. Conclusions

Here, we have explored the anticancer properties of a set of salicylaldehyde benzoylhydrazone with methoxy substituents on both rings. Based on the derived results, we can draw the following conclusions.

The designed dimethoxy hydrazones show promising molecular characteristics and promising properties for lead and drug similarity. The cytotoxicity data revealed that dimethoxy hydrazones in the series had significantly stronger growth-inhibitory effects on leukemic suspension cell cultures and demonstrated lower anticancer activity on human breast adenocarcinoma MCF-7 cells and triple-negative MDA-MB-231 breast adenocarcinoma. 4-methoxysalicylaldehyde benzoylhydrazones are more active than the corresponding 3-methoxysalicylaldehyde benzoylhydrazones on bcr-abl+ chronic myeloid leukemia cell lines BV-173, K-562, and AR-230 and T-cell leukemia SKW-3. 4-methoxysalicylaldehyde-3-methoxybenzoylhydrazone (compound **4**) and 4-methoxysalicylaldehyde-4-methoxybenzoylhydrazone (compound **5**) showed activities on SKW-3 cells in nanomolar concentrations.

Dimethoxy hydrazones exhibited low activity towards the healthy HEK-293 cells, indicating tumor selectivity. 3-methoxysalicylaldehyde benzoylhydrazones are less toxic and more selective than the corresponding 4-methoxysalicylaldehyde benzoylhydrazones. 3-methoxysalicylaldehyde-2-methoxybenzoylhydrazone (compound **1**) and 3-methoxysalicylaldehyde-4-methoxybenzoylhydrazone (compound **3**) are non-toxic up to 100 μM, showing their remarkable tumor selectivity. Among the tested compounds, 3-methoxysalicylaldehyde-4-methoxybenzoylhydrazone (compound **3**) is the most promising anticancer agent against leukemic K-562, SKW-3, and HL-60 cells, with high selectivity indices between 36 and 42.

As the tested compounds showed high activities on bcr-abl+ chronic myeloid leukemia cell lines BV-173, K-562, and AR-230, human cAbl tyrosine kinase was explored as a probable protein target. The compounds fit well in the binding site and form stable complexes with the protein for 1 μs MD simulation.

## Figures and Tables

**Table 1:** Structures of the designed methoxysalicylaldehyde methoxybenzoylhydrazones.

** 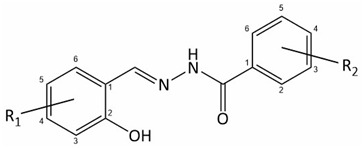 **	**ID**	**R_1_**	**R_2_**
	1	3-methoxy	2-methoxy
	2	3-methoxy	3-methoxy
	3	3-methoxy	4-methoxy
	4	4-methoxy	3-methoxy
	5	4-methoxy	4-methoxy

**Table 2:** Physicochemical properties and pharmacokinetic parameters of the methoxysalicylaldehyde methoxybenzoylhydrazones. *Mw*—molecular weight, *pK_a_* value, *f_A_*—fraction of the ionized molecules, *logP*—partition coefficient, *logD*_7.4_—distribution coefficient at pH 7.4, *PSA*—polar surface area, *FRB*—count of free rotatable bonds, *HBDs*—hydrogen bond donors, *HBAs*—hydrogen bond acceptors, *R5*—count of the violations from Lipinski’s Rule of 5, *water solubility*, *GI absorption*—gastrointestinal absorption, *oral BA*—oral bioavailability, INSATU—unsaturation, *BA score*—bioavailability score, *BBB permeability*—blood–brain barrier permeability, *CYP inhibition*—inhibition of CYP enzymes, *P-gp substrate*—substrate of P-gp, *drug likeness*, *lead likeness*, *synthetic accessibility*, *f_u_*—fraction of the unbound to plasma protein molecules, *CL*—total clearance, *VD_ss_*—steady-state volume of distribution, *t*_1/2_—half-life.

Property	1	2	3	4	5
Mw g/mol	300.31	300.31	300.31	300.31	300.31
pK_a_	8.40	8.39	8.47	8.01	8.08
f_A_	0.09	0.09	0.08	0.20	0.17
logP	3.31	3.75	3.45	3.86	3.56
logD_7.4_	3.24	3.71	3.38	3.77	3.42
PSA Å	80.15	80.15	80.15	80.15	80.15
FRB	6	6	6	6	6
HBDs	2	2	2	2	2
HBAs	6	6	6	6	6
R5	0	0	0	0	0
Water solubility	Moderately soluble	Moderately soluble	Moderately soluble	Moderately soluble	Moderately soluble
GI absorption	High	High	High	High	High
Oral BA	INSATU	INSATU	INSATU	INSATU	INSATU
BA score	0.55	0.55	0.55	0.55	0.55
BBB permeability	No	No	No	No	No
CYP inhibition	No	No	1A2	1A2, 2C9	No
P-gp substrate	No	No	No	No	No
Drug likeness	Yes	Yes	Yes	Yes	Yes
Lead likeness	Yes	Yes	Yes	Yes	Yes
Synthetic Accessibility	2.72	2.72	2.62	2.69	2.63
f_u_	0.016	0.013	0.015	0.012	0.014
CL L/h/kg	0.45	0.43	0.44	0.39	0.43
VD_ss_ L/kg	0.208	0.206	0.208	0.209	0.211
t_1/2_ h	0.32	0.34	0.32	0.37	0.34

**Table 3:** IC_50_ values (µM) ± standard deviations of the salicylaldehyde benzoylhydrazones. The anticancer activities of the compounds were measured on five leukemia cell lines (BV-173, K-562, SKW-3, AR-230, and HL-60), one breast ER+ adenocarcinoma cell line (MCF-7), and one TNBC cell line (MDA-MB-231). IC_50_s below 1 µM are given in bold. Selectivity was evaluated against normal human embryonic kidney cells, HEK-293.

Cell Line	1	2	3	4	5
HL-60	11.2 ± 2.1	3.6 ± 0.3	2.4 ± 0.1	2.1 ± 0.2	4.7 ± 0.5
BV-173	33.5 ± 3.4	2.7 ± 0.2	2.7 ± 0.2	2.2 ± 0.3	1.4 ± 0.2
K-562	20.4 ± 2.9	2.1 ± 0.1	2.8 ± 0.4	1.6 ± 0.2	1.5 ± 0.1
AR-230	50.5 ± 6.6	5.9 ± 0.7	>100	4.5 ± 0.4	1.1 ± 0.2
SKW-3	4.3 ± 0.5	1.4 ± 0.1	2.7 ± 0.3	**0.8 ± 0.1**	**0.5 ± 0.1**
MCF-7	30.1 ± 3.2	25.6 ± 2.8	36.1 ± 4.4	23.3 ± 2.7	21.3 ± 1.9
MDA-MB-231	22.0 ± 2.8	26.9 ± 3.3	23.5 ± 3.4	19.8 ± 3.1	60.1 ± 5.0
HEK-293	>100	16.8 ± 2.3	>100	8.6 ± 1.5	10.4 ± 1.1

**Table 4:** Ligand efficiencies (*LEs*) and selectivity indices (*SIs*) of the methoxysalicylaldehyde methoxybenzoylhydrazones. The anticancer activities of the compounds were measured on five leukemia cell lines (BV-173, K-562, SKW-3, AR-230, and HL-60), one breast ER+ adenocarcinoma cell line (MCF-7), and one TNBC cell line (MDA-MB-231). Selectivity indices (*SIs*) above 10 times and the corresponding *LEs* are given in bold.

Cell Line	1		2		3		4		5	
	*LE*	*SI*	*LE*	*SI*	*LE*	*SI*	*LE*	*SI*	*LE*	*SI*
HL-60	0.225	>9	0.247	5	0.255	**>42**	0.258	4	0.242	2
BV-173	0.203	>3	0.253	6	0.240	**>19**	0.257	4	0.266	7
K-562	0.213	>5	0.258	8	0.252	**>36**	0.263	5	0.265	7
AR-230	0.195	>2	0.238	3	>0.182	>1	0.243	2	0.271	9
SKW-3	**0.244**	**>23**	**0.266**	**12**	**0.253**	**>37**	**0.277**	**11**	**0.286**	**21**
Avg. leukemia	0.216	>8	0.252	7	0.237	**>27**	0.260	5	0.266	9
MCF-7	0.206	>3	0.209	1	0.202	>3	0.211	0	0.212	0
MDA-MB-231	0.212	>5	0.208	1	0.210	>4	0.214	0	0.192	0
Avg. breast cancer	0.209	>4	0.209	1	0.206	>4	0.213	0	0.202	0
Avg. all cancers	0.214	>7	0.240	5	**0.228**	**>20**	0.246	4	0.248	7
HEK-293	>0.182	- *	0.217	- *	>0.182	- *	0.230	- *	0.226	- *

* not applicable.

**Table 5:** Docking ChemPLP scores and ClemPLP scores per unit Mw of imatinib and methoxysalicylaldehyde methoxybenzoylhydrazones.

Compound	ChemPLP Score	ChemPLP Score/Molecular Weight
Imatinib	122.312	0.248
1	70.373	0.234
2	71.499	0.238
3	73.266	0.244
4	71.747	0.239
5	67.669	0.225

## Data Availability

Data is contained within the article (and Supplementary Materials).

## Supplementary Materials

The following supporting information can be downloaded at https://www.mdpi.com/article/10.3390/molecules30051015/s1, Figure S1: The top-scored docking poses of imatinib and the designed dimethoxy hydrazones (**1**–**5**) to the Abl tyrosine kinase are depicted. Ligands are presented in element-colored sticks, while water molecules are shown in ball-and-stick representation. Figure S2: The Cα RMSDs (top), RMSFs (middle), and RMSDs of ligands’ heavy atoms (bottom) of the studied complexes during the production dynamics are presented. Figure S3: The intramolecular hydrogen bonds of compounds **4** (at 218.4 ns) and **5-a** (at 801.3 ns) are depicted, while the poses of the remaining compounds **1** (at 642.9 ns), **2** (at 556.2 ns), **3** (at 752.1 ns), and **5-b** (at 358.5 ns) are shown for comparison. In derivatives **1**–**3**, the methoxy group is adjacent to the hydroxy group, and the hydrogen atom is oriented towards the neighboring oxygen atom of the methoxy group at distances varying between 2.067 and 2.24 Å. In compounds **4** and **5**, the methoxy group is shifted by one position, and the hydroxyl hydrogen atom is oriented toward the linker’s electronegative nitrogen and oxygen atoms for hydrogen bond formation. Figures S4–S18: IR and NMR spectra of the dimethoxy hydrazones.
