# Supplementary figures and images for: Salicylaldehyde Benzoylhydrazones with Anticancer Activity and Selectivity: Design, Synthesis, and In Vitro Evaluation
Source: Molecules. 2025 Feb 22;30(5):1015. doi: 10.3390/molecules30051015 (PMC11901818; doi:10.3390/molecules30051015)

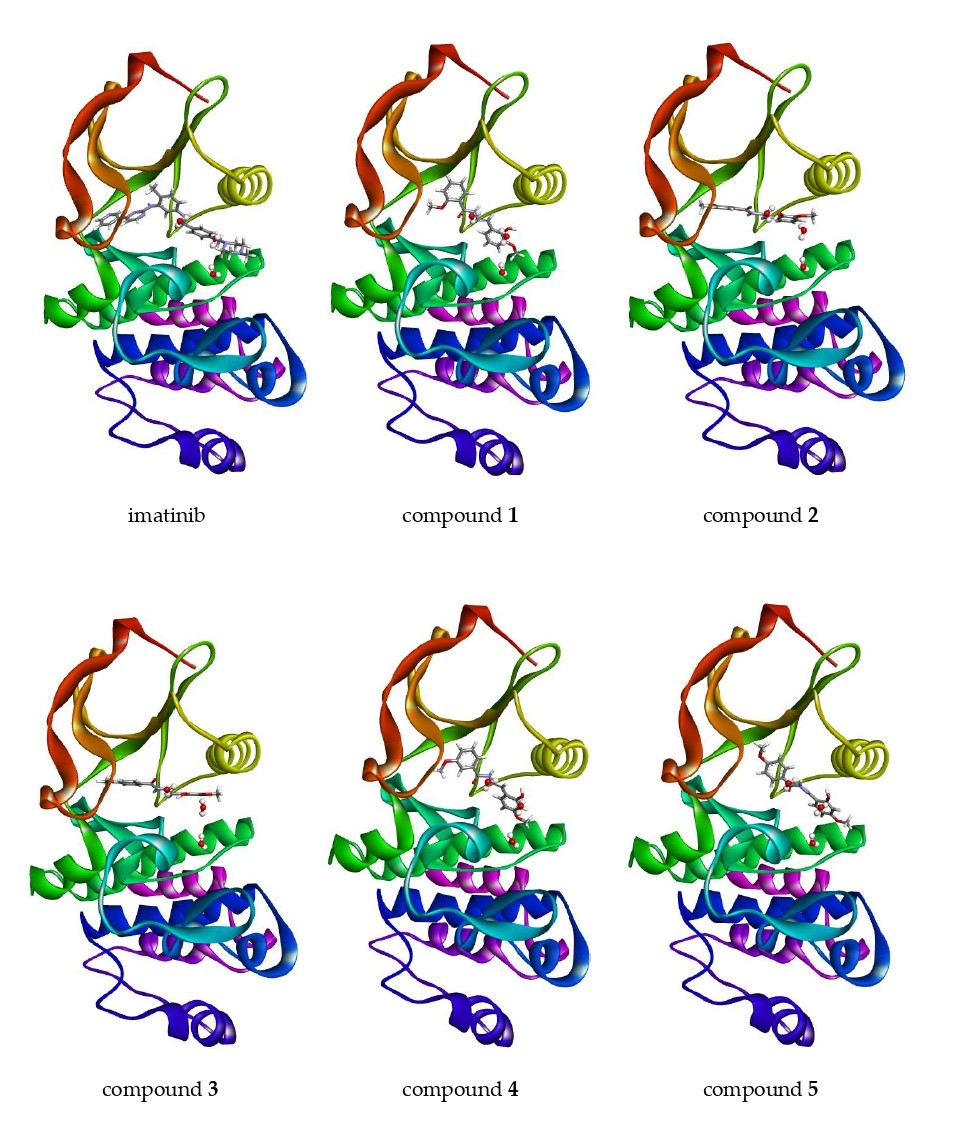

Supplement: Supplementary file 1 [file molecules-30-01015-s001.zip › Figure S1.jpg]

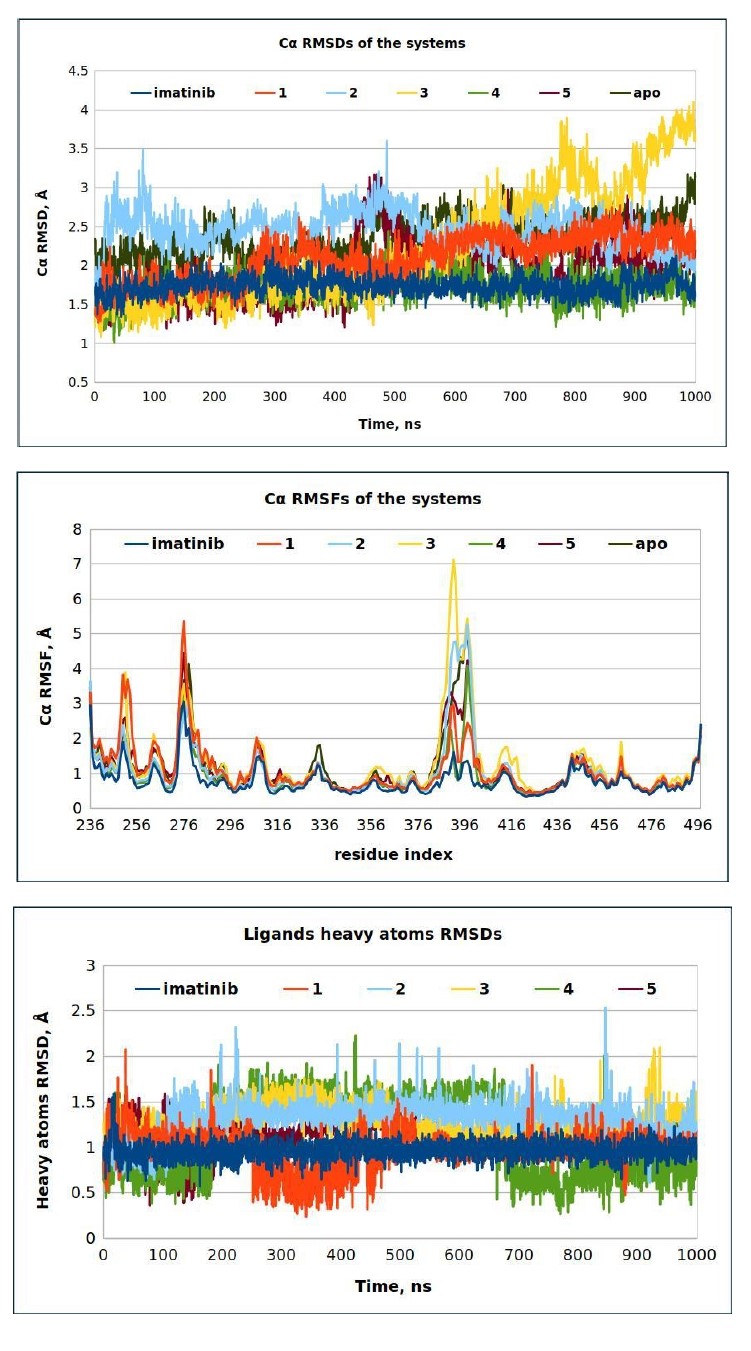

Supplement: Supplementary file 1 [file molecules-30-01015-s001.zip › Figure S2.jpg]

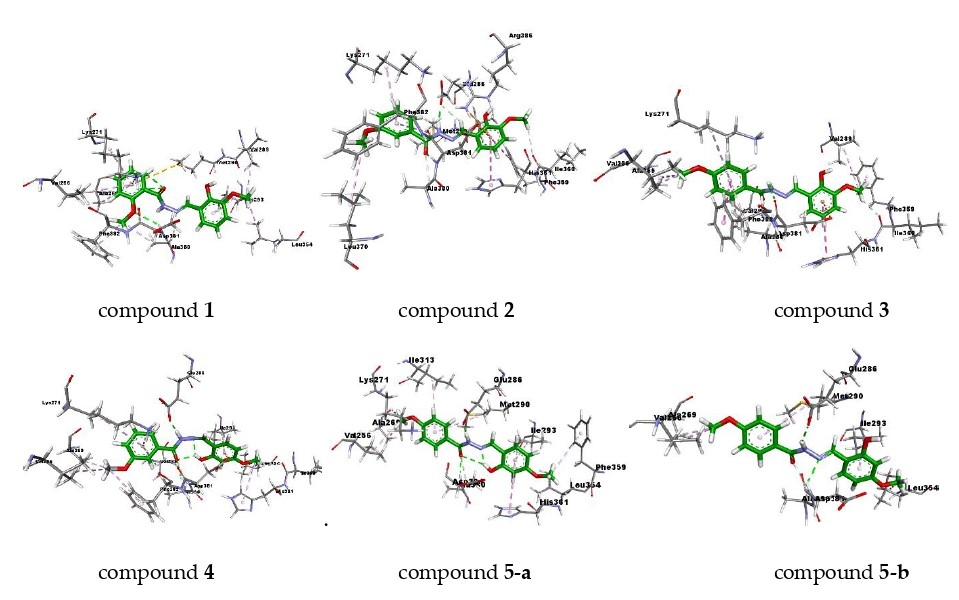

Supplement: Supplementary file 1 [file molecules-30-01015-s001.zip › Figure S3.jpg]
